# Advancements in microalgae-mediated technologies for antibiotic removal from wastewater: a review

**DOI:** 10.1007/s10532-026-10273-2

**Published:** 2026-03-17

**Authors:** Neha Pathania, Swati Kumari, Kamlesh Thakur, Saurabh Kulshrestha, Rohit Khargotra

**Affiliations:** 1 Faculty of Applied Science and Biotechnology, Shoolini University of Biotechnology & Management Sciences, Bajol-Solan, HP 173212 India; 2 Escuela Politécnica Superior, Universidad Francisco de Vitoria, Ctra. Pozuelo-Majadahonda Km 1.800, 28223 Pozuelo de Alarcón, Madrid, Spain; 3 Dean R&D, Bennett University, Plot No. 8-11, Tech Zone II, Greater Noida , Uttar Pradesh 201310 India

**Keywords:** Antibiotics, Wastewater treatment, Microalgae, Photocataysis, Photodegradation

## Abstract

The development of efficient systems for removing antibiotics from wastewater is being pushed by increase in antibiotic resistance brought on by the environmental discharge of antibiotics. Antibiotic were not completely eliminated using conventional technologies like activated sludge, constructed wetland systems and many other procedures. A viable alternative for treating wastewater through adsorption, accumulation, biodegradation, photodegradation, and hydrolysis has recently been investigated using microalgae-based technology. This review focuses on effects of antibiotics on microalgae, as well as the ways in which microalgae remove antibiotics and how they work with other technologies to do so, including photocatalysis, advanced oxidation, and complementary microorganism degradation. The physiochemical and operational parameters like pH, temperature, light intensity and many more which influence the elimination of antibiotics in the wastewater treatment system. Future research requirements, further opportunities, the limitations of the available microalgae-based technologies were also discussed.

## Introduction

**Fig. 1:**
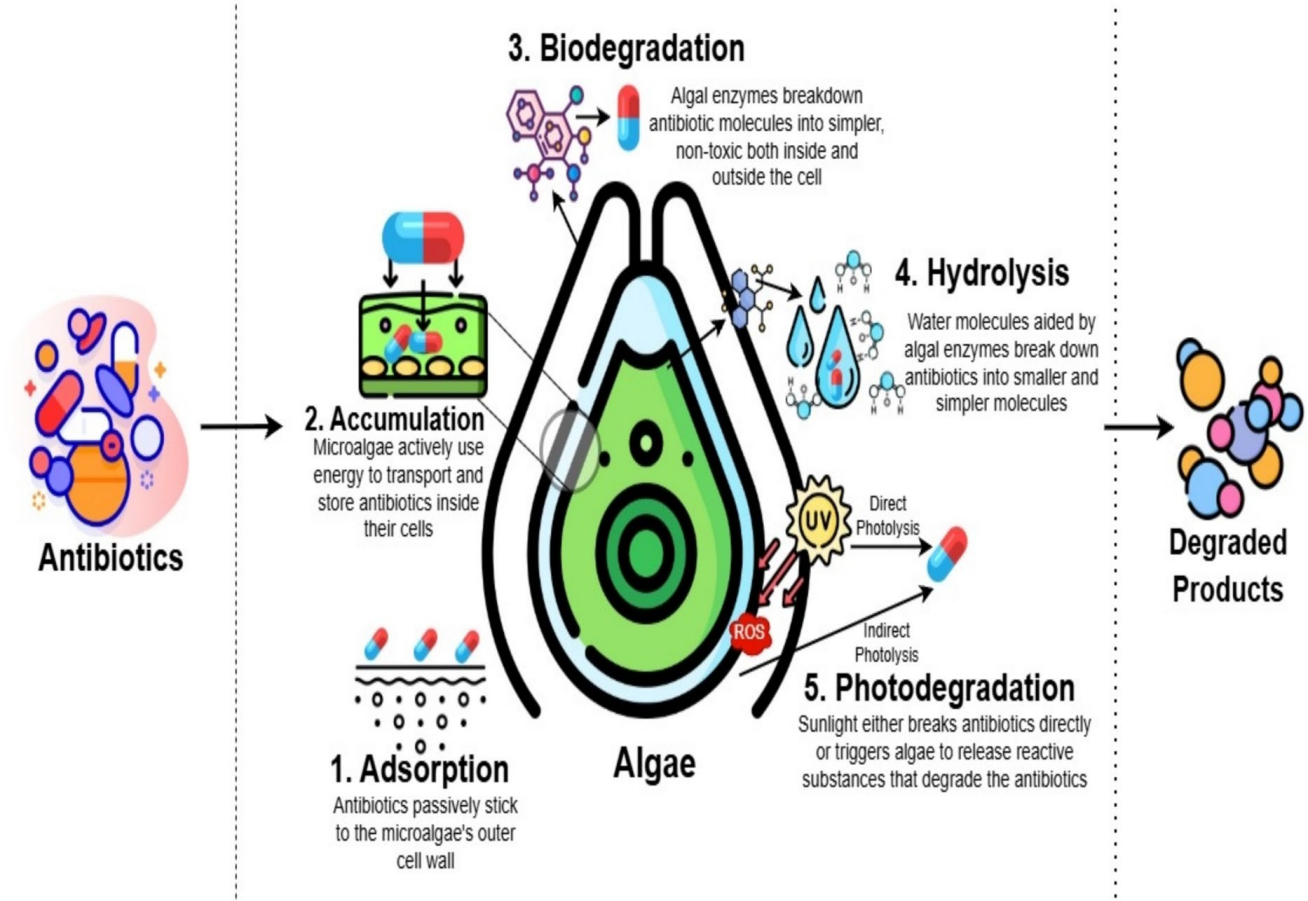
Mechanism of antibiotic degradation by microalgae including processes like adsorption, accumulation, biodegradation, and hydrolysis and photo degradation

Water is an essence of life and something that almost all living things require. However, because of the numerous evolutionary ways of contamination that result from various advances in technology made over the years, this resource is becoming increasingly scarce in its pure state. Water pollution is any alteration to the physical, chemical, or biological properties that has a detrimental effect on people or animals. Numerous micro-biological organisms, including bacteria, viruses, and protozoa, are present. It might lead to a number of water-borne illnesses. Water is directly used by humans and animals for a variety of reasons, but the cleanliness of the water is vital. It may have a direct impact on health as a result (Briggs 2003). Antibiotics are chemicals that have antibacterial, antifungal, or antiparasitic activity. They are frequently used to treat and prevent illnesses that are infectious in both humans and animalsin the cattle industry for reasons of promoting development (Kümmerer 2009). According to Lai et al. (2017) and Rizzo et al. (2013), wastewater treatment plants (WWTPs) are one of the main places where ecological exposure to antibiotics occurs. To encourage the emergence and extent of antibiotic-resistant bacteria (ARB) and antibiotic-resistant genes (ARGs), a substantial evolutionary force could act on human and microbial community as a result of antibiotic abuse and misuse (Ashbolt et al. 2013 and Ying et al. 2017). Recently microalgae-based technology has gained a major attention as an effective technique to treat industrial and municipal effluent with its advantages including pollutant removal, CO_2_ fixation and the possibility to discover some goods from algae. Because of their versatility and ability to endure harsh environments, microalgae are intriguing candidates for better wastewater treatment. They also guarantee the low-cost removal of heavy metals (Zeraatkar et al. 2016), emerging contaminants (ECs) (Sutherland and Ralph 2019), and a variety of nutrients. The current review focuses on the most recent developments in the removal of antibiotics using microalgae. Future research perspectives are also considered, as well as the current research limitations in this field (Fig. 1).

## Mechanism of antibiotics removal by microalgae

**Fig. 2:**
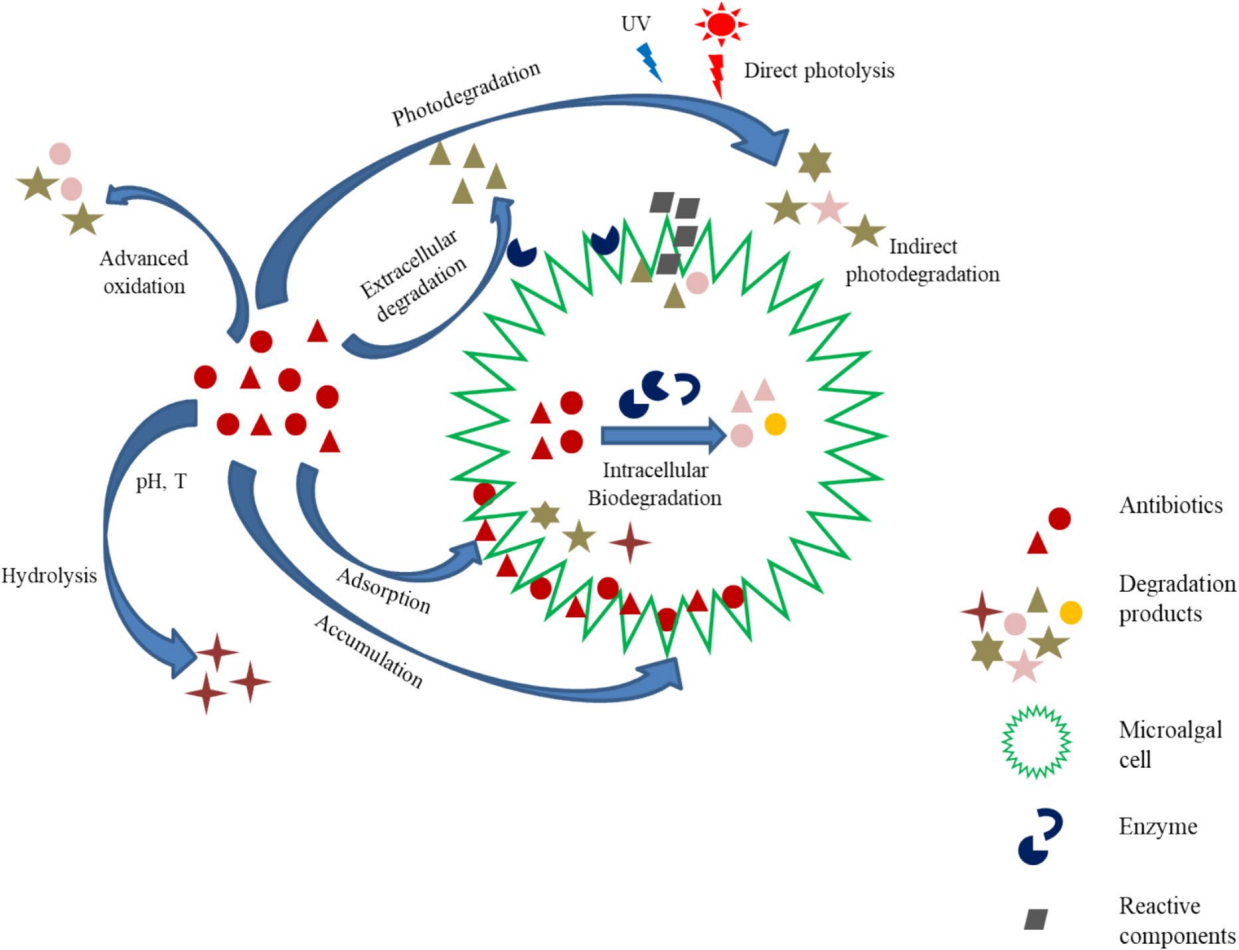
Various degradation pathways of antibiotics in wastewater treatment processes

There are various degradation pathways of antibiotics in this processes, some are biotic mechanism that occurs outside the cell (extracellular) and other can be abiotic that occurs inside the cell itself (intracellular) contributing to the antibiotics removal as shown in Fig. 2.

### Adsorption

When pollutants are passively attached to a solid substrate, the process is referred as adsorption (Bai and Acharya 2016) Many studies on the removal of antibiotics utilising adsorbents such activated carbon, nanomaterials and biochar have been appeared. The distribution of antibiotic ions or soluble compounds between the extracellular polymeric substances (the bio-polymers which are secreted by the microorganisms) of microalgae and the liquid phase occurs passively. The two kinds of EPS—bonded EPS, associated to the cells of microalgae, and free EPS, expelled by microalgae in interruption—both offer potential sites for the adsorption of pollutants (Fomina and Gadd 2014). Microalgae may function well as an antibacterial adsorbent. This process involves some interactions like hydrophobic forces and electrostatic attraction, due to which antibiotics adsorbs onto the negatively charged microalgal cell surfaces or EPS which do not even require the cellular metabolism. Some components have major impact on the efficiency like the microalgal species; the antibiotic concentration, temperature (20–30 °C) and pH (6–9); elevating the surface area for adsorption due to the smaller cell size present. For example, Chlorella vulgaris attains 100% removal of metronidazole via adsorption (Long et al. 2024).

Some studies shows very high adsorption rates of ciprofloxacin (91.5%) by species like *Auxenocholreallaprotothecoides*, with adsorption rate of 37.2–49.3% in other species like *Tetradesmusobliqus* and *Chlamydomonasacidophila* (Li et al. 2024). This process produces valuable biomass for biofuel or feed, very much cost-effective and unites very easily with fungi or bacteria consortia for better removal. It also marks the antibiotic pollution from sources like hospitals, lowering the antimicrobial resistance risk (Inuwa et al. 2023).

The adsorption, which is a non-cellular process, is being carried out by the functional groups and polymer accumulation on the algal cell walls (Davis et al. 2003; Xiong et al. 2018). When dead algal biomass is used to take up the antibiotics, the effectiveness of the adsorption process can be evaluated. However, the performance of the adsorption process varies greatly depending on the particular antibiotic due to the structural, functional, and hydrophilicity differences between and microalgae. Therefore, when the antibiotics have a charge different to that of the microalgae around them, it is preferable that they be more hydrophobic and hydrophilic.

The elimination of tetracycline from high-rate algal ponds (HRAP) was likewise discovered to be mostly mediated by adsorption (deGodos et al. 2012; Norvill et al. 2017). According to Ahmed et al. (2016) and Tan et al. (2015), the key mechanisms by which antibiotics are adsorbed onto microalgae biomass include many forces and bonding like H-bonding, electrostatic forces and the hydrophobic effect. Adsorption plays the role of initial step in the antibiotic’s removal using the microalgae as further steps can be accumulation and biodegradation and it’s not even the primary method of the antibiotic’s degradation (Yu et al. 2017a, b).

Adsorption frequently focuses antibiotics on the carriers/sludge, which enhance their concentration and makes its available for microbial biodegradation in wastewater treatment system. Some case studies show this type of synergy essentially in biofilm and activated sludge system. Examples include sulphonamides, β-lactams and fluroquinolones (Li and Zhang 2010).

Activated sludge from saline and freshwater along with the batch reactors treated 11 antibiotics which includes cephalexin (97.2% removal rate) by biodegradation and ampicillin (37.8–58.6%) by adsorption using limited biodegradation at 100 µg/L (Li and Zhang 2010).

Biofilm-Granular Activated Carbon (BGAC) combined biodegradation and adsorption for antibiotics like ampicillin (AMP), penicillin G (PCG) and cephalosporin C (CPC) which are β-lactam antibiotics. When granular activated carbon adsorbs antibiotics, high efficiency removal occurs after the growth of biofilms with 8 mg/L AMP having direct correlation with biodegradation rates and AMP-degrading bacteria.

Some antibiotics like norfloxacin (NFX), roxithromycin (RTM) and sulfamethazine (SMZ) exposed to Chlorella vulgaris at 100 µg/L first adsorbs to extracellular membrane and then bioaccumulates intracellulary before its degradation to water and its derivatives (Long et al. 2024).

Fluoroquinolones like norfloxacin (NOR), ofloxacin (OFL) and ciprofloxacin go through rapid adsorption (42–90%) followed by degradation (upto 40.7%) whereas sulfadiazine (SDZ) and sulfamethoxazole (SMX) goes for primary biodegradation (22.5–53.3%), in saline sludge due the availability of divalent cation which enhances degradation and lowers down the adsorption.

### Accumulation

According to Bai and Acharya (2016) and Davis et al. (2003), the process of removing pollutants from water occurs intracellularly. Adsorption is a passive process that doesn’t use energy, whereas accumulation is active, uses energy, and moves more slowly. Microalgae are able to take in and store contaminants for use during growth processes in the cell, along with nutrients. Algae accumulation have a very prominent role in the termination of antibiotics like doxycycline, trimethoprim and sulfamethoxazole (Bai and Acharya 2017; Prata et al. 2019).

Unlike adsorption (physical–chemical binding of antibiotics to the microalgal cell walls/EPS, accumulation internalizes antibiotics, which leads to further degradation or intracellular storage. Factors like less antibiotics and salinity (NaCl promotes levofloxacin up tale in *Chlorella vulgaris*).

Chlorella vulgaris accumulated levofloxacin (82.35% removal) and azithromycin with intracellular processes. Recent studies also shows that accumulation shares 20–65% removal of quinolones, sulfamethoxazole and trimethoprim in species like Scenedesmusobliquus (Li et al. 2022a, b, c, d).

Reactive oxygen species, that is important for regulating cellular metabolism at normal amounts but can cause serious cell damage or even death at elevated concentrations, can be produced by some accumulating antibiotics (Xiong et al. 2018) whereas, the algal cell’s metabolism might mitigate the consumption of antibiotics.Because of this, accumulation takes place before biodegradation, and two further mechanisms cooperate inside algal cells to hasten the absorption of certain antibiotics. Reactive oxygen species, that basically regulate cellular metabolism in microalgae, are produced inside the cell by some bio-accumulated medicines at low concentrations (Xiong et al. 2018).

### Biodegradation

According to several studies (Ke et al. 2010; Naghdi et al. 2018), biodegradation refers to the method by which helps the antibiotics to dissolve intracellularly and extracellularly, where many of those derivatives being broken down are then consumed by the algal cells. In this procedure, which involves the antibiotics degradation in three steps, ceftazidime was first adsorbed on algae, and then transported through the algal cell wall, and finally broken down by algal enzymes (Yu et al. 2017a, b).

Microalgae remove the emerging contaminants from the aqueous phase as well as from biomass using a variety of mechanisms, but one of the most efficient is biodegradation. Bioaccumulation and adsorption of compounds that biodegrade in the intracellular or intercellular phase, respectively, of the biomass. According to Naghdi et al. (2018), the extracellular cells help in conversion of some harmful antibiotics into very low or not toxic products, that is then biodegraded and accumulated by intracellular enzymes. Metabolic and co-metabolic degradation are some patterns of algal metabolism for the degradation of the antibiotics, in which co-metabolism degradation has non-specific enzymes to break down them with some additional fuel and carbon required, on the other hand, metabolic degradation have only antibiotics as the only source of energy and fuel (Table 1).

**Table 1:** Removal efficiency of antibiotics classes vs different microalgal species

Antibiotic Class	Microalgal species	Removal efficiency	Technique	References
Beta-lactam (e.g., Amoxicillin, Cefalexin)	Unspecified algae (lipid-extracted biomass)	30.5–33.6%; 71.2 ± 38.9%	Biodegradation; Biosorption	Zhang et al. (2023)
Multiple (33 antibiotics, incl. various classes)	Coelastrum sp.	5–50% higher than activated sludge	Biosorption/Biodegradation	Zhang et al. (2023)
Various classes	Chlorella variabilis	Up to 62.3% overall	Bioaccumulation/Photodegradation	Zhang et al. (2023)

### Photodegradation

**Fig. 3:**
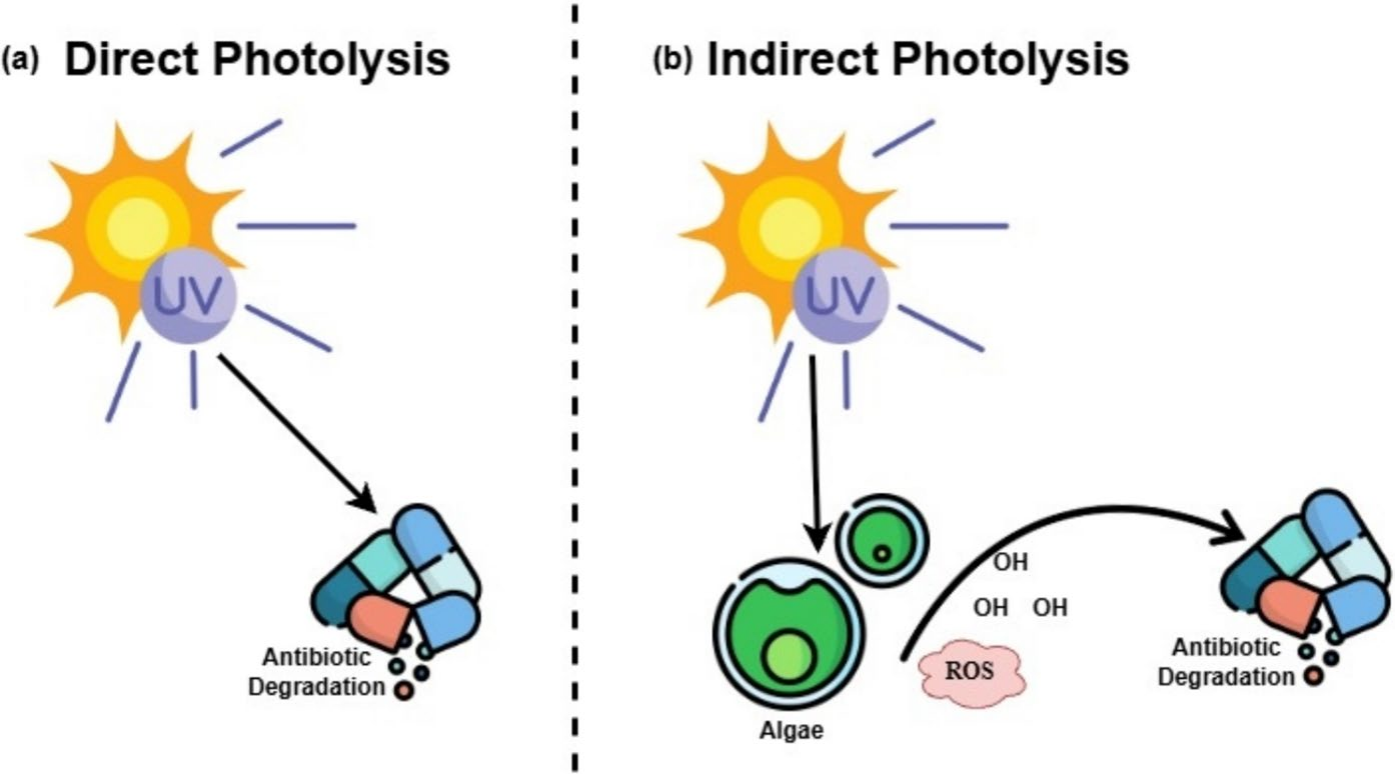
Photodegradation of algae by **a** Direct Photolysis where UV light hit an antibiotic molecule directly causing it to break and **b** Indirect Photolysis where UV light hits algae, which then produces Reactive Oxygen Species (ROS) or hydroxyl radicals which then attack the antibiotic

This method works well, is sustainable, affordable, and simple to improve. Its indirect photodegradation is triggered by reactive components that algae make when light is present as shown in Fig. 3b while direct photolysis is provided by UV light as shown in Fig. 3a. When UV light is there and without algae, photolysis can directly remove antibiotics in higher amount (Chen et al. 2016; Jiang et al. 2018). In several wastewater treatment systems involving microalgae, tetracycline, ciprofloxacin, cefazolin, cephapirin were also eliminated by direct photolysis, according to reports from deGodos et al. (2012), Bai and Acharya (2017).

When there are algae in the process, indirect photodegradation enhances the removal of some antibiotics. The antibiotics degrade when reactive oxygen species, like hydroxyl radicals, are produced through indirect photodegradation from algal components as shown in Fig. 3b. In *Nannochloris* sp.-mediated culture, Bai and Acharya (2017) reported ciprofloxacin and triclosan were completely eradicated after a week of incubation and a full day of radiation using a photolysis process. According to Liu et al. (2004), photolysis degradation is accelerated by free radical production, which increases as microalgae concentration increases. Number microalgae boost up rate at which norfloxacin was photo-biodegraded. Levofloxacin was exposed to *S. obliquus* throughout a treatment process, but no photodegradation was noticed.

### Hydrolysis

Antibiotics are present in the pharmaceutical wastewater and contaminate it so hydrolysis is another method which helps in the elimination of antibiotics; it helps in breaking down the antibiotics in to certain smaller molecules. Enzymatic hydrolysis is one of its types which is brought by algal metabolites or enzymes and can also be considered as a kind of biodegradation. Once the antibiotic is completely immersed in the water, its polarity and hydrophobicity are enhanced by the interactions that make the degradation of antibiotics by microalgae much easier (Wang et al. 2018). Sulphaguanidine and sulfadiazine are two antibiotics which sustained its hydrolytic activity of pH approx4.0. Another study showed that tetracycline does not hydrolyse properly when treated with microalgae (Norvill et al. 2017) whereas fluroquinolones and sulphonamides are those antibiotics which exhibit resistance to it.

Hydrolysis contributes to antibiotic removal in microalgae-based wastewater treatment as an abiotic process involving water-mediated cleavage of chemical bonds, often enhanced by alga;-induced pH or enzymes. It primarily affects antibiotics with hydrolysable groups like β-lactam rings, breaking them into less toxic byproducts, and accounts for 5–35% of total removal alongside biosorption and photolysis.

54.52% of sulfadiazine removed by hydrolysis along with biodegradation by *Chlamydomonas* species (Singh et al. 2024).

Due to the presence of strained four-membered β-lactam ring, antibiotics particularly β-lactams like cephalosporins and penicillin are highly favourable to hydrolysis, which undergoes nucleophilic attack by the ions present in the water, which leads to low antimicrobial activity (Mitchell et al. 2014).

There is a major role of algal enzymes in enzymatic hydrolysis as there are some microalgal enzymes which help in exceeding abiotic rates by 20–50% which includes some intracellular oxidoreductases and extracellular hydrolases, catalyse enzymatic hydrolysis, cleaving β-lactam rings or ester bonds after the accumulation process (Frascaroli et al. 2024a, b) (Table 2).

**Table 2:** Mechanisms by which microalgae remove antibiotics from wastewater vs their efficiency

Mechanism	Description	Typical efficiency	Key factors/examples	References
Bioaccumulation	Active uptake and intracellular accumulation	22–62% (e.g., total antibiotics by consortia)	Membrane transport; energy-dependent	Zhang et al. (2023)
Biodegradation	Enzymatic breakdown into metabolites	5–96% (e.g., erythromycin by Chlorella pyrenoidosa)	Extracellular enzymes; microalgae-bacteria synergy	Zhang et al. (2023)
Biosorption	Passive adsorption onto algal cell surfaces via ion exchange or complexation	30–100% (e.g., 100% for metronidazole by *Chlorella vulgaris)*	Surface functional groups; rapid process	Zhang et al. (2023)
Algae-Bacteria Consortia	Synergistic degradation via oxygen supply and pH shifts	5–8% improvement over monocultures (e.g., 53.9 mg/L/day COD boost)	Community interactions; broader spectrum	Kiki et al. (2023)
Photodegradation	Light-induced breakdown, often minor role	5–35% contribution (e.g., ciprofloxacin, ofloxacin)	UV light; pH conditions	Li et al. (2022a, b, c, d)

## Factors influencing microalgal antibiotic removal efficiency

### Physicochemical properties

The chemical and structural characteristics of microalgae also has a key role in how well they eradicate the antibiotics present like their molecular weight, charge, functional groups and hydrophobicity are the important factor which affects elimination of antibiotics. The rate of removal is enhanced by the biosorption process as some of the antibiotics like clarithromycin are adsorbed outside the cell due to their favourable conditions with the cell wall functional groups (Frascaroli et al. 2024a, b; Zhang et al. 2023).

The affinity for microalgal interfaces and sensitivity to degradation is usually resolved by the antibiotic’s speciation and the degree of ionization, which is affected by pH of solution. Zwitter ion production is promoted by some of the antibiotics that are adsorbed at pH values (Li et al. 2022a, b, c, d). Therefore, physiochemical balance between the microalgal system and the antibiotic present is equally important for the treatment process (Zhang et al. 2023).

### Parameters (light, pH, temperature, HRT, etc.)

These are some of the environmental factors and parameters which plays major role in elimination of the antibiotics:

**pH:** Recent studies shows that when the optimal pH values between 7.5 and 8 exist then the removal efficiency is at its best peak, also it affects the rates of degradation and adsorption of the antibiotics (Li et al. 2022a, b, c, d).

**Temperature:** Most of the microalgae show temperature range between 25 and 30 °C, which also affects the kinetics of removal and growth rate as well as metabolic activities and enzymatic activity of the microalgae (Li et al. 2022a, b, c, d).

**Light intensity and quality: **For optimizing the removal rates of the antibiotic, intensity, quality and light wavelength all are very essential as the metabolic activity and growth of the antibiotic breakdown is directly connected with the photosynthesis which also depends on light for microalgae (Li et al. 2022a, b, c, d).

**Hydraulic retention time (HRT):** The elimination of antibiotics and other contaminants is influenced by the amount of time period water remains in the system. Shorter period of HRT can enhance the algal development while longer one boosts up the removal rate of the antibiotic (Frascaroli et al. 2025).

**CO**_**2**_** enrichment: **Proper microalgal growth is enhanced by sufficient amount of CO_2_, i.e., 5–16% which also then enhances the removal rate of antibiotics (Li et al. 2022a, b, c, d).

### Assessing and adapting microalgal strains

The most important thing to keep in mind is the species of microalgae chosen for the treatment process. Different strains have different kind of bioaccumulation, biosorption and biodegradation. Physiological reactions related to strain, like pigment overproduction and stress adaptation, can improve removal efficiency even further (Frascaroli et al. 2024a, b). Furthermore, in long-term uses, certain microalgae can increase their resilience and removal efficiency by gradually adapting to the existence of antibiotics (Frascaroli et al. 2025).

In conclusion, a complex interaction between the physicochemical features of the antibiotics, the treatment system’s operational parameters, and the innate abilities and adaptability of the chosen microalgal strains determines the effectiveness of microalgae-mediated antibiotic removal (Zhang et al. 2023).

## Removal of antibiotics using additional enhanced hybrid methods

### Microalgae—microorganism consortia

**Fig. 4:**
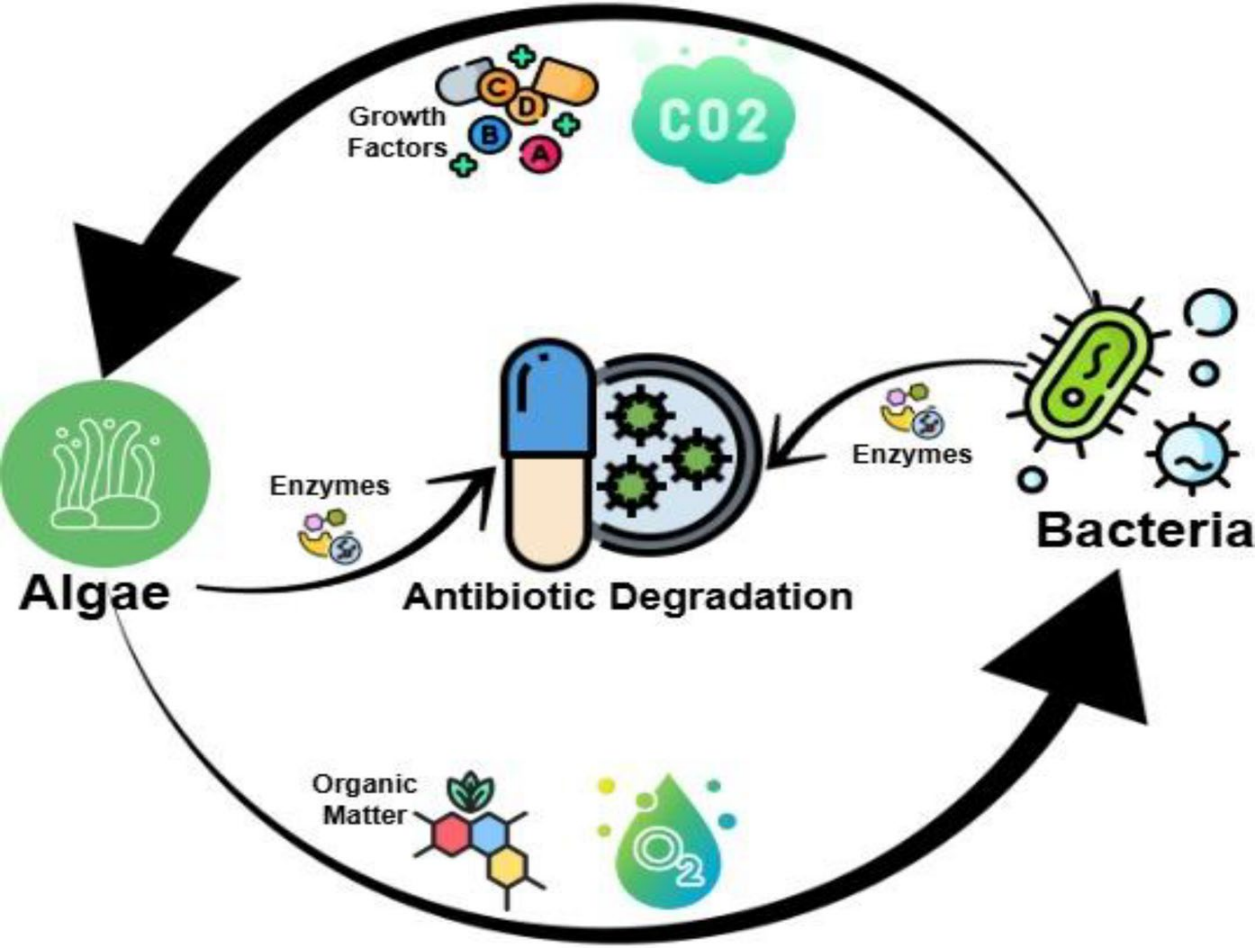
Schematic representation of Algae bacteria symbiosis where algae produce O_2_ and organic matter for the bacteria, while the bacteria produce CO_2_ and growth factors (like Vitamin B12) for the algae and both organisms contribute enzymes to the process of antibiotic degradation

Microalgae-bacteria systems exploit mutualistic connections to promote antibiotic removal as shown in Fig. 4.

Mechanisms: Photodegradation (light-driven processes), combined biodegradation and biosorption (cell surface/EPS adsorption) (Li et al. 2022a, b, c, d).

## Advantages:

- Chlorella sp. paired with bacteria achieves > 97% removal of cephalosporins via synergistic metabolism (Li et al. 2022a, b, c, d).
- High-rate algal ponds (the ponds that use microalgae to treat the wastewater) remove > 93% tetracycline through photodegradation and biosorption at hydraulic retention times (HRT) of 4 days (Li et al. 2022a, b, c, d).
- Eliminates external aeration needs by generating oxygen via photosynthesis, reducing energy costs (Mohammed et al. 2023).

### Microalgae-advanced oxidation processes

These are a pre-treatment step designed to enhance the efficacy of treating the water that contains resistant organic chemicals, such antibiotics. Due to their high removal efficiency and quick removal rates, AOPs have been widely used in wastewater treatment systems. Oxidation mechanisms may remove the antibiotics directly during the AOPs-algae therapy. Indirect photodegradation brought on by AOP can also get rid of antibiotics. For instance, Fe (III) that is photochemically reactive improves the clearance of antibiotics by microalgae by causing the development of reactive oxygen species. The introduction of Fe(III) can also speed up the photodegradation of norfloxacin by microalgae (Zhang et al. 2012). AOPs, however, have detrimental effects on microalgae when used in conjunction with microalgal treatment, including UV-induced mutation and death from elevated H2O2 or Fe(II) concentrations. Consequently, future studies should focus on determining the particular circumstances that are appropriate for every microalgal therapy.

Advantages:

They play a vital role for many pollutants and operational needs as it provide a number of noteworthy benefits in water and wastewater treatment applications.

Key advantages:

- **Rapid reaction times:** In contrast to conventional chemical or biological processes, AOPs enable rapid breakdown of contaminants because of the high oxidation potential and non-selective nature of hydroxyl radicals, which leads to significantly shorter retention durations.
- **Broad-spectrum pollutant removal:** Numerous organic pollutants that are frequently resistant to traditional treatments, such as dyes, insecticides, medications, and volatile organic compounds (VOCs), can be efficiently broken down by AOPs.
- **Full organic mineralization:** Pollutants are converted into stable inorganic compounds, such as water, carbon dioxide, and salts.
- **No toxic byproducts:** Unlike other chemical treatment methods (like chlorine disinfection), AOPs don’t introduce any more hazardous substances. They lessen the generation of toxic byproducts.
- **Compact system design**: Due to its great efficiency, which permits smaller reactors and footprints, AOPs are suitable for installations with restricted space.
- **Disinfection capability**: Certain AOP systems remove organic contaminants and pathogens from water simultaneously by disinfecting it with UV or ozone components.
- **No sludge generation:** AOPs simplify disposal and save operating expenses because they don’t produce sludge like some chemical and biological processes do.
- **Effective heavy metal removal:** Beyond eliminating organic contaminants, AOPs can occasionally precipitate and remove heavy metals from water.

#### Ozonation process

Direct ozone oxidation and indirect ozone oxidation through the generation of free radicals are the two mechanisms by which ozone-based antibiotics degrade (Eq. (1)) (Kasprzyk-Hordern et al. 2003). As a 2electrophilic reactant, ozone may efficiently eliminate the antibiotics TC, SM, and QN by targeting their aromatic rings and unsaturated double bonds (Wang and Law 2013).

1 $$ {\mathrm{3O3}} + {\mathrm{H2O}} \to {\mathrm{2OH}} + {\mathrm{4O2}} $$

Furthermore, large amounts of OH can be produced by the ozone oxidation process in conjunction with hydrogen peroxide (H2O2), ultraviolet (UV), or catalysts (Eqs. (2)–(6)). This OH can then be utilized to degrade organic pollutants (Ling et al. 2020). The H2O2/ozone coupling system significantly raised the CTC clearance rate in cow effluent from 30 to 65%.

2 $$ {\mathrm{O}}_{{3}} + {\mathrm{H}}_{{2}} {\mathrm{O}}_{{2}} \to {\mathrm{OH}} \cdot + {\mathrm{O}}_{{2}} + {\mathrm{HO}}_{{2}} $$

3 $$ {\mathrm{H}}_{{2}} {\mathrm{O}}_{{2}} \to {\mathrm{HO}}_{{2}} - + {\mathrm{H}} $$

4 $$ {\mathrm{H}}_{{2}} {\mathrm{O}}_{{2}} \to {\mathrm{HO}}_{{2}} - + {\mathrm{H}} + $$

5 $$ {\mathrm{O}}_{{3}} + {\mathrm{O}}_{{2}} - \to {\mathrm{O}}_{{3}} - + {\mathrm{O}}_{{2}} $$

6 $$ {\mathrm{O}}_{{3}} - + {\mathrm{H}}_{{2}} {\mathrm{O}} \to {\mathrm{OH}} \cdot + {\mathrm{OH}} - + {\mathrm{O}}_{{2}} $$

#### Fenton process

Reagents in this reaction, namely H_2_O_2_ and Fe^2+^ iinteract to produce OH radicals (Eq. (7)), that breaks down the antibiotics (Rozas et al. 2016) (Table 3).

7 $$ {\mathrm{Fe}}^{{{2} + }} + {\mathrm{H}}_{{2}} {\mathrm{O}}_{{2}} \to {\mathrm{Fe}}^{{{3} + }} + {\mathrm{OH}} + {\mathrm{OH}} - $$

**Table 3:** Principal disadvantages and benefits of AOPs used in wastewater treatment

Processes	Benefits	Disadvantages
Fenton process	(Vintage season) No pH changes are necessary	Inorganic sludge production
	Colour removal from the wastewater	Consumption of highly energetic photons produced by UV lighting artificially
	Biodegradability improvement	Energy use and chemical use
	Toxicity reduction	Complexity of the technology
	High efficiency	Lack of sufficient experience at full scale
	Catalyst can even be activated by sunlight	
Ozone	Colour removal	Low performance for COD removal
	Sludge production-free	Complexity of the technology
	Enhancing biodegradability	
	High phenol compound removal	
	Lowering of toxicity	

#### Ultraviolet (UV) irradiation

This is a persistent disinfection phase in wastewater treatment which enhances the removal of antibiotics through both direct and indirect photodegradation. Via direct photolysis, UV exposure can destroy antibiotics that are sensitive to light. When they are exposed to UV light, it absorbs photons that can break down their bonding proteins (Liu et al. 2017). According to Yuan et al. (2011), doxycycline degrades quickly when exposed to UV radiation. With UV irradiation, more than 74% of the cefradine was eliminated (Du et al. 2015). Indirect photodegradation of antibiotics can be accelerated adding UV irradiation to microalgae treatment. According to Curtis et al. (1994), UV light rapidly attenuates in wastewater at a depth of several centimetres to decimetres; therefore, indirect photodegradation may be the main route for antibiotic elimination. Microalgae were crucial to the biodegradation process, while UV light was used to start the process. *S. obliquus* demonstrated the best clearance (99.84%) of cefradine and amoxicillin within 24 h of treatment. Liu et al. (2017) introduced a secondary step of the treatment to eliminate ceftazidime after treating it with UV-microalgae. This improved hybrid system’s removal effectiveness.

### Activated sludge system

This method has been traditionally used over past 100 years to treat the municipal sewage water. Because it requires less energy, and cost-effectiveness for reprocessing, the hybrid one is moderately viable for the water treatment (Gonçalves et al. 2017; Quijano et al. 2017). According to Kümmerer (2009), the family of cephalosporins includes over 60% of all antibiotics used for medicinal purposes. Guo and Chen’s (2015) discovery shown that when antibiotics were primarily treated with microalgae, they avoid directly coming in touch with the bacteria. When compared to the initial cephalosporin concentration of 100 mg/L, its hybrid system demonstrated very high eradicating capacity (up to 97.8%). This method is an effective way to treat the water and guard against the bacterial community gaining resistance, creating ARBs, or releasing ARGs into the environment.

This system depends on microbial flocs for adsorption of tetracyclines/fluoroquinolones (50–80%) and aerobic degradation of sulphonamides (> 85%). Longer period of solid retention time (SRT > 20 days) enhances the elimination by 20–50% by the help of slow growing degraders (Li and Zhang 2010).

Our knowledge of the basic mechanisms underpinning the elimination of micro pollutants is still lacking because of the complex, and advanced interconnections that exist between the bacteria and microalgae. In order to develop a hybrid system with many applications, highest pollutants removal efficiencies, efficient biomass productivity, high yields of value-added by-products, and biofuel at comparatively very lesser cost, genetic modification can also help select the right strains of bacteria and microalgae.

### Constructed wetland technique

This technique is also widely used to clean wastewater from industry, government, and agriculture. These systems have effectively and successfully eliminated many of the different contaminants, including antibiotics and ARGs. It’s an environmentally conscious substitute to conventional treatment methods since they are less expensive to build, run, and maintain. However, they may also show poor efficiency and long retention duration at low temperatures (Liu et al. 2019a, b).

The process of nitrification is frequently the rate-limiting stage because there is usually barely any oxygen accessible in CWs (Xiong et al. 2018). The DO generated by microalgae during photosynthesis might reimburse for any potential drawbacks in this method, improving the elimination of pollutants in CWs-microalgae systems.

### Microalgae-adsorbent integration

Algae-derived biochar offers sustainable, high-efficiency adsorption and catalytic properties:

- **Biochar production**: Pyrolysis of algal biomass creates nitrogen-rich, porous structures ideal for antibiotic adsorption (e.g., π–π interactions, ion exchange) (Tan et al. 2023; Ghosh et al. 2025).
- **Fe (III)-modified biochar:** Improves photocatalytic degradation of sulfonamides under light exposure (Tan et al. 2023).
- **Nanocomposites:** Algal biochar combined with TiO_2_ or graphene oxide enhances oxidative degradation of tetracyclines) (Tan et al. 2023; Ghosh et al. 2025).
- **Nutrient enrichment**: Adding phosphorus or CO_2_ to biochar-algal systems boosts metabolic activity and antibiotic uptake (Li et al. 2022a, b, c, d).

There are some significant risks associated with biomass disposal when TPs or residual antibiotics accumulate, due to enhancement of antimicrobial resistance (AMR) and environmental contamination, which can also affect water bodies, soil microbes, human health via food (Polianciuc et al. 2020) There are some strategies for safe biomass handling like composting (microbial degradation under optimal conditions like pH, temperature control), incineration (calcify TPs and antibiotics at high temperature but needs energy input and regulatory input) and further treatment (AOPs degrades residuals before disposal, hybrid methods and on farm strategies are some more treatment) (Andraskar et al. 2023; Zhou et al. 2023a, b; Haque et al. 2024).

**Fig. 5:**
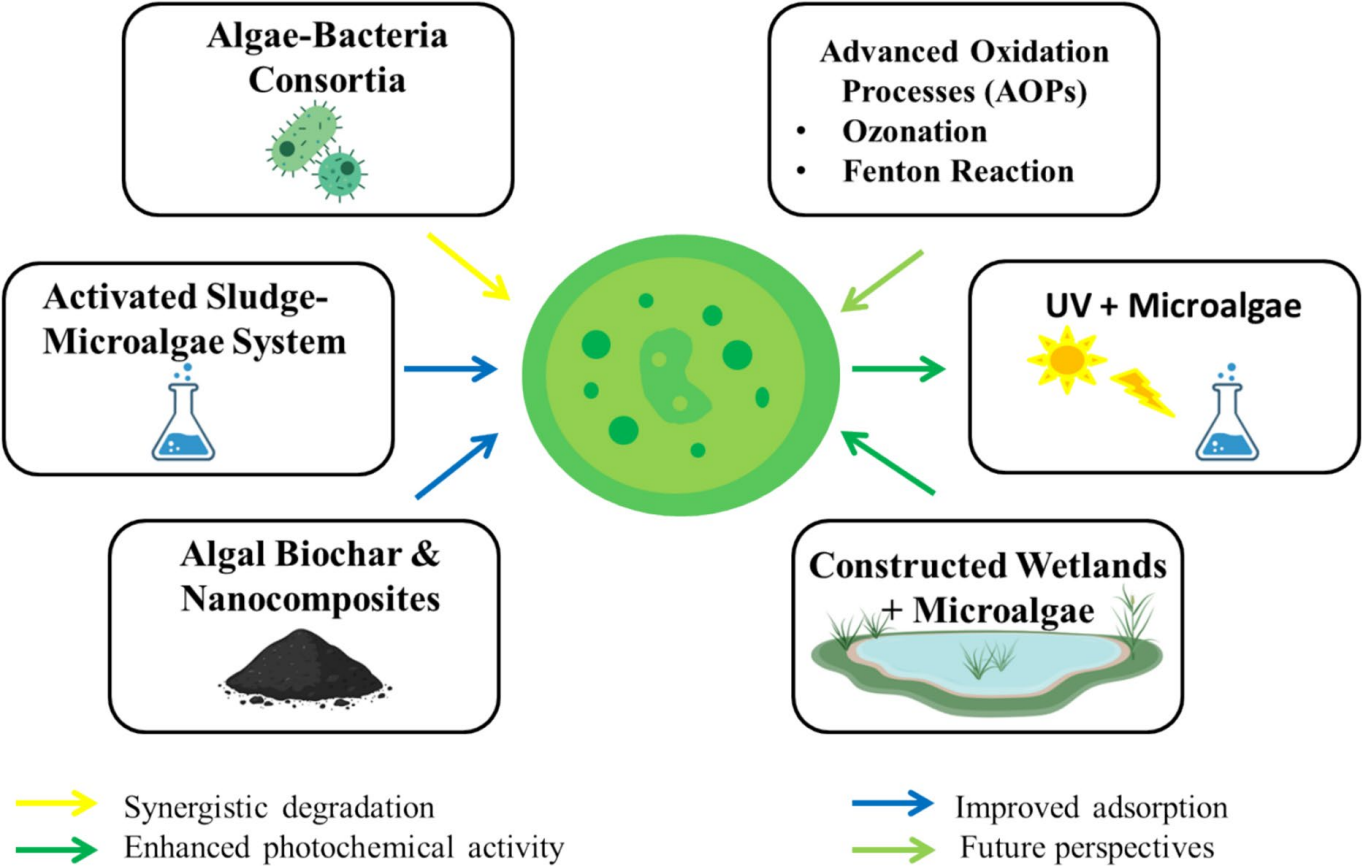
Schematic representation of hybrid and integrated approaches combining microalgae with bacteria, advanced oxidation processes, activated sludge, UV, constructed wetlands, and biochar/nanocomposites for enhanced antibiotic removal from aquatic systems

These hybrid and integrated approaches address limitations of standalone systems, offering scalable, energy-efficient solutions for antibiotic removal from the wastewater as shown in Fig. 5 (Mohammed et al. 2023; Li et al. 2022a, b, c, d).

Most of the research focuses the lab/small scale system with limited study on pilot scale microalgae setups for antibiotic removal.

Recent study showed the treatment of wastewater spiked with antibiotics (olfloxacin, ciprofloxacin, trimethoprim, clarithromycin ~ 50 μg/L each) using a bioreactor trial of Auxenochlorellaprotothecoides. This large scale system achieved 45.7–70% of antibiotic removal in 3–4 days. It highlighted some challenges like potential biomass but no specific location, costs or capacity were discussed. (Kiki et al. 2023) (Table 4).

**Table 4:** Performance of microalgal systems for antibiotic removal across lab vspilot scales

Scale	Description	Typical efficiency	Key examples	References
Lab Scale	Small-volume (50–2250 mL) batch experiments with optimized light, temperature, and media	70–100% (e.g., 93% tetracycline by *Chlorella vulgaris* in 4 days; 99% with acetate addition)	*Chlorella pyrenoidosa* (99% oxytetracycline, 5 days); *Nannochloris sp.* (32%, 14 days)	Xiong et al. (2025)
Pilot Scale	Larger bioreactors (e.g., continuous supply with synthetic/real wastewater, 3–4 days retention)	45–70% (e.g., 45.8–70.1% multiple antibiotics by *Auxenochlorellaprotothecoides)*	Long-term trials with swine manure wastewater; outdoor diurnal tests	Frascaroli et al. (2024a, b)

## Toxicity and challenges

### Toxic effects of antibiotics and transformation products on microalgae

Antibiotics like erythromycin, sulfamethoxazole, and tetracycline inhibit microalgal photosynthesis by disrupting electron transport in Photosystem II (PSII) and reducing chlorophyll fluorescence yields (Fv/Fm*Fv*/*Fm*) (Roy et al., 2021; Aderemi et al. 2021, b). For example: Erythromycin suppresses carbon assimilation and photophosphorylation in *Raphidocelissubcapitata*, reducing photosynthetic efficiency by 17–20% at environmentally relevant concentrations (10–50 µg/L) (Roy et al., 2021; Aderemi et al. 2021, b).

Transformation products (TPs) of sulfonamides and fluoroquinolones exhibit higher toxicity than parent compounds. TP126, a sulfamethoxazolebyproduct, causes acute and chronic toxicity to green algae (Zhang et al. 2023).

**Oxidative stress is a key mechanism**: antibiotics like ofloxacin and ciprofloxacin induce reactive oxygen species (ROS), overwhelming antioxidant defenses (e.g., catalase, glutathione S-transferase) and damaging cellular components) (Roy et al., 2021; Aderemi et al. 2021, b).

Processes like adsorption, accumulation and biodegradation diminish the transformation product (TP) toxicity and often decompose into less harmful products like biomass components and CO_2_ (Zhang et al. 2023) Using some enzymatic activities like oxidoreductases and laccases, microalgae further degrade antibiotic TPs as described in methylparaben studies in which Chlorella sorokiniana attain degradation and detoxification at the same time (Morillas et al. 2015). During primary biosorption phases, TPs accumulates in microalgal biomass, especially lipophilic ones but long term buildup by metabolism inside the cell (Yang et al. 2025).

### Impact on microalgal growth and metabolism

**Growth inhibition**: Antibiotics reduce specific growth rates by 30–60% in species like *Chlorella vulgaris* and *Auxenochlorellaprotothecoides* at concentrations > 10 µg/L. For instance, amoxicillin (20 µg/L) lowers biomass production by 40% compared to controls (Frascaroli et al. 2024a, b).

**Metabolic shifts**: Microalgae adapt by switching to heterotrophic metabolism under stress. *A. protothecoides* enhances pigment production (e.g., chlorophyll *B* by 37%) and acidifies its environment (pH drops by three units) to degrade antibiotics like sulfamethoxazole (Frascaroli et al. 2024a, b).

**Protein synthesis disruption**: Tetracycline and kanamycin reduce protein content by 50–70% in *Dictyosphaeriumpulchellum*, impairing enzymatic and structural functions (Bashir and Cho 2016).

As discussed above AOPs break down complex molecules into smaller one, which are more reactive, TPs helps in further reduction through processes like adsorption, accumulation and biodegradation. TPs generated during AOPs and photodegradation may show higher level of toxicity towards the microalgae as compared to the original ones and with some temporary environmental risks, influenced by biological activities.

## Recent advancements and novel approaches in microalgae-mediated antibiotic removal

### Genetic engineering of microalgae

CRISPR/Cas9 and other gene-editing tools are being used to enhance microalgal antibiotic degradation capabilities:

**Stress tolerance**: Engineered *Auxenochlorellaprotothecoides* exhibits improved resilience to high antibiotic concentrations (up to 100 µg/L) while maintaining > 70% removal efficiency for sulfamethoxazole and ciprofloxacin (Frascaroli et al. 2024a, b).

**Degradation pathways**: Overexpression of cytochrome P450 enzymes in *Chlamydomonasreinhardtii* accelerates the breakdown of erythromycin and tetracycline by 40% compared to wild strains (Li et al. 2022a, b, c, d).

**Heterotrophic adaptation**: Modified *Chlorella vulgaris* strains switch to mixotrophic metabolism under low-light conditions, enhancing antibiotic uptake and biodegradation rates (Xiong et al. 2021).

### Use of omics technologies to decipher mechanisms

Multi-omics approaches are clarifying removal pathways and stress responses:

**Metagenomics**: Revealed that algal–bacterial consortia in constructed wetlands reduce tetracycline resistance gene (*tetW*) abundance by 56% compared to bacterial-only systems (Wang et al. 2022).

**Transcriptomics**: Identified upregulated genes in *A. protothecoides* under antibiotic stress, including those involved in ROS scavenging (e.g., *sod1*, *cat2*) and cell wall biosynthesis (Frascaroli et al. 2024a, b; Zhang et al. 2023).

**Metabolomics**: Detected 12 intermediate products of sulfamethoxazole degradation by *Scenedesmusobliquus*, linking biodegradation to glutathione conjugation pathways (Eheneden et al. 2023a, b; Li et al. 2022a, b, c, d) (Table 5).

**Table 5:** Hybrid system and Standalone membrane with their advantages as well as antibiotic removal efficiency

System type	Description	Typical antibiotic removal efficiency	Advantages	References
Hybrid (Microalgae-Bacteria)	Algae-activated sludge or consortia (e.g., HMAS with LEDs) enhancing enzymatic breakdown	70–96% (e.g., 75% SCOD; 5–50% higher than standalone for multi-class antibiotics)	Oxygen supply from algae; wider spectrum; no aeration needed	Mohammed et al. (2023)
Hybrid (Microalgae-Membrane)	MPBR integrating filtration for biomass retention	Up to 90% + nutrients/antibiotics (doubled biomass vs standalone PBRs)	Independent HRT/SRT control; higher retention	Goh et al. (2022)
Standalone Microalgae	Pure algal cultures (e.g., *Chlorella vulgaris*) in photobioreactors using biosorption/bioaccumulation	30–99% (e.g., 93% tetracycline; limited to specific classes)	Simpler operation; biomass production	Xiong et al. (2021)

## Opportunities and future directions

### Breeding and enhancement of strains

Enhancing the removal efficiency of microalgae is a key research priority. Selective breeding, adaptive evolution, and genetic engineering can yield strains with higher tolerance to antibiotics and their transformation products, as well as improved biosorption, bioaccumulation, and biodegradation capabilities. For example, *Auxenochlorellaprotothecoides* has shown promising results, efficiently removing multiple antibiotics even under high-stress conditions without growth inhibition (Frascaroli et al. 2024a, b; Li et al. 2022a, b, c, d). Many antibiotics classes can be degraded by some organisms which also have specialization in transforming the products; it builds consortia with many of the bacteria or fungi which might then enhance the clearance rates (Xiong et al. 2021). To accelerate the process of microalgae-based treatment, it needs further more research into resilient and new strains (Zhang et al. 2023).

### Enhancement of operational environments

The efficacy of the antibiotics degradation and the microalgae growth both are affected by some of the factors like light intensity, pH, temperature, and the availability of the nutrition. Biomass productivity and the contaminants removal can be improved by the dilution rates in photo bioreactors, which also favour the microalgal development and the enhanced nitrogen removal efficiency. There are some statistical tools which can help in determining the best conditions for various regions and times of the year; one of those is research surface methodology (RSM). For more successful implementation, some operational strategies might be tailored to the environmental conditions and wastewater properties.

### Highly advanced integrated therapy methods

Integrated and hybrid systems are very important for further growth. Some of the techniques have shown enhanced removal efficiency and operational stability like algae-activated sludge, artificial wetlands and algae-bacteria consortia. There are certain methods like biosorption, photodegradation and biodegradation which are used to remove certain antibiotics by more than 90–95% using hybrid constructed wetlands and high-rate algal ponds (HRAPs) (Li et al. 2022a, b, c, d). Also, there are some other adsorbents and catalysts which help in enhancing the removing efficiency of prolonged antibiotics and some of the transformed products. Advanced oxidation processes (AOPs) are more often helpful as it is used in the initial stage of the treatment because it increases the biodegradability of antibiotics and then ease their further eradication by microalgae (Xiong et al. 2021). So, in conclusion, further phase of the antibiotic’s removal depends on some of the factors like developing the working conditions, enhancing the strains, anticipating toxicity and some more presiding issues. All these phases are then supported by ongoing innovation and work so that these advanced techniques would be very important to progress the results into efficient, long-lasting and effective wastewater treatment systems as they hold promising approaches to manage the variety of the wastewater streams (Table 6).

**Table 6:** Antibiotic type, microalgal strain, system configuration, and their removal efficiency

Antibiotic type	Microalgal strain	System configuration	Removal efficiency (%)	References
Ofloxacin (OFL)	Auxenochlorellaprotothecoides	Batch culture, BG-11 medium, 50 μg/L initial	61	Frascaroli et al. (2024a, b)
Sulfamethoxazole (SMX)	Chlorella pyrenoidosa	Batch culture, BG-11 medium, 5 mg/L initial	49	Huang et al. (2024)
Ciprofloxacin (CIP)	Auxenochlorellaprotothecoides	Batch culture, BG-11 medium, 50 μg/L initial	70	Frascaroli et al. (2024a, b)
Tetracycline (TC), Oxytetracycline (OTC), Chlortetracycline (CTC)	C. vulgaris with C. rosea (co-culture)	Batch, wastewater medium, 0.25 mg/L initial	94–99	Huang et al. (2024)
Metronidazole (MDZ)	Chlamydomonasacidophila	Batch culture, BG-11 medium, 50 μg/L initial	33	Frascaroli et al. (2024a, b)
Sulfamethoxazole (SMX)	Scenedesmusquadricauda	Batch culture, BG-11 medium, 5 mg/L initial	16	Huang et al. (2024)

## Summary and conclusion

Utilizing microalgae for wastewater treatment could provide a potentially sustainable way to reduce antibiotic contamination and antibiotic resistance risk in the aquatic environment. The mechanisms of antibiotics removal by microalgae include biodegradation, biosorption, bioaccumulation and photodegradation. Compared with traditional wastewater treatments, microalgal approaches are generally more cost-effective to operate which require less energy and maintain better environmental compatibility (Bai & Acharya 2016; Yu et al. 2017a, b; Li et al. 2022a, b, c, d). Recently, increases in the antibiotic removal rate have been reached by combining microalgae with bacterial communities and applying advanced oxidation technologies which includes ozonation and Fenton-type oxidation (Mohammed et al. 2023; Zhang et al. 2024). Hybrid systems may thus offer a promising way to more consistently to treat complex wastewaters containing assortments. Several important knowledge gaps are yet to be addressed: for example, long-term effects, toxicity and environmental risks of antibiotic transformation by-products considering environmental and operating condition variability because some are strain-specific and their assessment with respect to pilot-scale studies. Hybrid systems also require continuous techno-economic and life cycle analyses so that comprehensive advantages and disadvantages compared with the other technologies Li et al. (2022a, b, c, d) and Xiong et al. (2021). In addition with dynamics of antibiotic resistance genes in algae and hybrid systems require further study to create proper treatment strategies and to assess regulatory acceptance of any such treatment (Li et al. 2022a, b, c, d; Xiong et al. 2021).

Future studies should be focus on the development of stress-tolerant and genetically engineered microalgal strains, optimization of physicochemical and operational parameters and application of multi-omics approaches like transcriptomics, metagenomics and metabolomics to unravel the resistance mechanisms and metabolic pathways involved in antibiotic removal. In the future, more emphasis will be placed on pilot-scale validation, hybrid system design and environmental risk assessment to translate the laboratory-scale successes into practical, eco-friendly wastewater treatment solutions supportive of sustainable water management and global public health goals.

## Data Availability

No datasets were generated or analysed during the current study.
